# Full- or Split-Thickness Skin Grafting in Scalp Surgery? Retrospective Case Series

**DOI:** 10.29252/wjps.8.3.331

**Published:** 2019-09

**Authors:** Carolina Maria Helena Hilton, Lisbet Rosenkrantz Hölmich

**Affiliations:** 1 Department of Oncology, Rigshospitalet, København Ø, Denmark;; 2 Lisbet Rosenkrantz Hölmich, Department of Plastic Surgery, Herlev Gentofte University Hospital, Copenhagen University, Herlev, Denmark

**Keywords:** Full thickness, Split thickness, Scalp surgery, Skin, graft, Transplant

## Abstract

**BACKGROUND:**

Closure of skin defects after scalp surgery may be accomplished by grafting; either split- or full-thickness. Both methods are used in Denmark, and the optimal approach on scalp defects without exposed bone is not known. This study aimed to investigate if the two methods were equal regarding graft take as primary outcome and as secondary outcomes complications and number of outpatient visits/ number of days from surgery until the last outpatient visit for the recipient site (as a proxy for time to healing), hypothesizing that they were.

**METHODS:**

The present retrospective single-center case series reported our experience using the two types of skin grafts after scalp surgery in the inclusion period from 1.1.2014 to 30.09.2015. Data were analyzed according to graft type with a full-thickness skin graft (FTSG-group) or a split-thickness skin graft (STSG-group).

**RESULTS:**

In the inclusion period, 106 patients had surgery (28 with a FTSG and 78 with a STSG). Irrespectively of which skin graft that was used, we found no statistically significant difference regarding percentage of adherence, complications or number of outpatient visits and time from operation until last outpatient visit regarding the recipient site (*p*>0.05).

**CONCLUSION:**

Our findings supported that use of either FTSG or STSG in scalp lesions were equal choices.

## INTRODUCTION

Scalp surgery for skin tumors often produces defects larger than what can be closed directly and therefore, reconstructive surgery is necessary. Multiple methods of reconstruction are available, including free flaps, local advancement flaps and skin grafting.^1^^–^^4^ Either a full-thickness skin graft (FTSG) or a split-thickness skin graft (STSG) may be employed. FTSGs are usually harvested from the groin, the upper inner arm and the supraclavicular fossa or, for scalp surgery, the adjacent scalp.^5^

STSGs are usually harvested from the upper anterior or lateral thighs, but almost any body region can be used if necessary. After harvest at the donor site, the graft is applied to the defect and a dressing is placed over to secure contact with the wound bed and prevent bleeding and formation of hematoma by providing slight compression. The dressing is held in place using either sutures or staples, and remains until the patient arrives for the first outpatient visit, typically on the fourth-seventh day after surgery.^5^

For scalp-surgery, including region-parietalis, region-occipitalis and the postauricular area, surgeons use either a STSG or a FTSG as opposed to the facial area or the hand where a FTSG is most commonly used due to cosmetic and functional reasons.^5^ Limited donor areas for FTSG can be a reason for using STSG. There are a number of advantages and disadvantages with the two types of grafts. The usage of a FTSG leaves the donor area to be closed by suturing, stapling or in rare cases with a split-thickness skin graft.^6^^-^^9^

FTSGs heal with a better cosmetic and functional result, give better coverage and have less secondary contraction, but require substantial nourishment, which leaves them vulnerable to insufficient vascularization.^6^^–^^9^ An inadequate wound bed is a major factor affecting especially FTSG take, which with its added thickness places higher demands regarding nourishment and revascularisation.^10^ STSGs have better initial take, larger donor site availability, and, in the scalp, contraction can be useful to decrease the size of the scar /alopecia.^10^

A STSG is harvested from the donor area with a dermatome (a surgical instrument used to produce thin slices of skin) and the donor area is left for re-epithelialization. A reason for using this type of graft for skin cancer patients is that they often present with recurring skin cancer, requiring more surgery and therefore it could be argued to leave their full skin graft reserves for tumors located in the face. In this study we did not consider the cosmetic aspect of what graft to use.^10^

The scalp is an area where the cosmetic and functional result is often of not as great importance as other areas, e.g. the face or hands. The possibility to mesh (to make holes in) this type of graft makes it able to cover larger areas than the size of the graft first harvested. Skin graft contraction occur in two stages; primary and secondary contraction. Primary contraction, which is greater in a FTSG, refers to the immediate reduction in size after harvest and secondary contraction, which is greater in a STSG, refers to wound bed contraction.^10^

This contraction can work in advantage for the patient over the long term by decreasing the grafted area over time. A relative contraindication exists for the use of a STSG over joints or where contractures must be avoided. However, it remains a necessary tool for larger defects, for example burn-wounds.^10^ Both types of grafts leave a difference in skin level, which is slightly more in STSG, and this graft is also more prone to dryness afterwards, since sebaceous glands are not transferred with the graft.^10^

There are no recommendations regarding which method is the most optimal to use on the scalp, and both are used in Denmark. The present study reported our experience with patients receiving the two types of skin grafts after scalp-surgery with excision of a skin tumor. The hypothesis was that the two methods were equal regarding graft take and complications.

## MATERIALS AND METHODS

The present study is a retrospective single-center case series including patients undergoing full-thickness or split-thickness skin grafting after scalp surgery for skin tumors during the period of 01.01.2014 and 30.09.2015. Patients were identified through the local hospital database using treatment codes for excision of a tumor on the head or neck, full thickness skin graft and split thickness skin graft. The study complies with the declaration of Helsinki. 

The primary outcome was percentage of skin graft adherence. Secondary outcomes were complications and number of outpatient visits/number of days from surgery until the last outpatient visit for the recipient site (as a proxy for time to healing). Results were analyzed according to FTSGs and STSGs. Inclusion criteria were surgery for a skin tumor in region-parietalis, region-occipitalis, the postauricular- or scalp-area leaving a defect that was either covered with a split-thickness or a full-thickness skin graft. 

If the patient had more than one surgery during the period, we only included the initial one, and in case of simultaneous tumors, the largest was included. A diversity of tumors was represented among the patients screened for inclusion. The diagnosis was not considered to influence what type of skin graft was used, nor the course of healing after excision of the tumor. We did not include region-frontalis in this study since this in many cases could be located in the face area rather than on the scalp, and a FTSG is almost always used in the facial area, if not a local flap.

Exclusion criteria were missing description of the primary lesion, missing follow-up to last planned outpatient visit or if the patient had a chronic wound after previous surgery in the area. Data on demographics, site of pathology, skin tumor diagnosis, ulceration or infection before surgery, longest excision diameter (as a proxy for excision area, since this was not always known), donor site location, length of hospital stay, complications, number of outpatient visits, time from operation until the last outpatient visit at the plastic surgery department, previous radiation therapy to the recipient area or chemotherapy within a month prior to surgery were obtained from hospital files. 

Infection before surgery was defined as cases where antibiotics were prescribed preoperatively. Data were analyzed according to grafting with a full-thickness skin graft (FTSG-group) or a split-thickness skin graft (STSG-group). Data are presented as number of patients and median (ranges). Fisher´s exact test and Chi-Square test were used for categorical data, Mann-Whitney U for ordinal or continuous data, and 95% confidence intervals as appropriate. A *p *value <0.05 was considered significant. All statistics were done using SPSS software (Version 21.0, IBM, New York, USA). 

## RESULTS

The medical records of 106 patients were reviewed. There were 28 patients receiving a full-thickness (FTSG-group) and 78 a split-thickness skin graft (STSG-group). Patient demographics were shown in Table 1. There were no differences with statistical significance between the two study groups. There was a trend towards more men getting FTSGs, smokers were more likely to get a STSG, people with previous radiation or recent chemotherapy got exclusively STSGs and larger defects were treated with STSG. 

**Table 1:** Patient demographics

	**FTSG-group** **(n=28) (%)**	**STSG-group** **(n=78) (%)**	***p*** ** value**
Age (years, range)	81 (59-93)	78 (37-104)	0.69
Gender			
Female Male	5 (17.9) 23 (82.1)	19 (24.4) 59 (75.6)	0.60
Smoking			
Yes No Previously	1 (3.6) 20 (71.4) 7 (25.0)	9 (11.5) 45 (57.7) 24 (30.8)	0.32
Alcohol abuse	5 (17.9)	12 (15.4)	0.77
Chronic Obstructive Lung Disease	2 (7.1)	4 (5.1)	0.65
Hypertension	21 (75.0)	43 (55.1)	0.08
Heart disease	9 (32.1)	21 (26.9)	0.63
Diabetes mellitus	2 (7.1)	3 (3.8)	0.61
Hypothyroidism	0 (0)	5 (6.4)	0.32
Immunosuppression	3 (10.7)	10 (12.8)	1.00
Radiation therapy Chemotherapy	0 (0) 0 (0)	5 (6.4) 1 (1.3)	0.32 1.00

Patient demographics in the full thickness skin graft group (FTSG-group) and the split thickness skin graft group (STSG-group)

Preoperative data, surgical information and tumor diagnosis are shown in Table 2. There was no significant difference in preoperative status of the patients with regard to infection or ulceration of the tumor before surgery. The FTSG-group had a median excision of 3.0 (1.5-5.0) cm and the STSG-group had a median excision of 4.0 (2.0-17.5) cm (*p*=0.001). There was a significant difference between the two groups regarding excision to galea fascia or pericranium. In the FTSG-group, 16 (57.1%) of the patients had an excision to the galea fascia and 12 (42.9%) to the pericranium as opposed to 24 (31.6%) and 52 (68.4%) patients in the STSG-group. For 84 (79.2%) of the patients, two of the following materials were used as inner layers in the dressing; euflavine, jelonet and nitrofuranzoin. 

**Table 2:** Preoperative data, surgical information and diagnosis

	**FTSG-group** **(n=28) (%)**	**STSG-group** **(n=78) (%)**	***p*** ** value**
Tumor with ulceration	14 (50.0)	47 (60.3)	0.38
Tumor region			
Regio-parietalis Regio-occipitalis Post-auricular area Scalp_a_	5 (17.9) 3 (10.7) 5 (17.9) 15 (53.6)	9 (11.5) 2 (2.6) 3 (3.8) 64 (82.1)	0.01
Longest excision (cm, range)	3.0 (1.5-5.0)	4.0 (2.0-17.5)	0.001
Excision to			
Galea fascia Pericranium Missing information	16 (57.1) 12 (42.9)	24 (31.6) 52 (68.4) 2	0.02
Donor site			
Split thickness, thigh Full thickness, clavicular area Full thickness, ear-area Full thickness, arm	19 (67.9) 2 (7.1) 7 (25)	78 (100.0)	
Transplant fixation			
Stapled Sutures	0 (0) 28 (100)	2 (2.6) 76 (97.4)	1.00
Inner dressing			
Euflavine Nitrofuranzoin Jelonet	4 (14.3) 22 (78.6) 24 (85.7)	7 (9.0) 64 (82.1) 67 (85.9)	0.75
Outer dressing			
Foam cloth Absorbent foam Missing information	11 (40.7) 16 (59.3) 1	27 (35.1) 50 (64.9) 1	0.01
Fixation of dressing			
Sutured Stapled	27 (96.4) 1 (3.6)	58 (74.4) 20 (25.6)	0.19
Diagnosis			
Basal cell carcinoma Squamos cell carcinoma Malignant melanoma	14 (50) 10 (35.7) 1 (3.6)	23 (29.9) 30 (39.0) 9 (11.7)	
Other tumors Missing information	3 (10.7)	15 (19.5) 1	

Preoperative data, surgical information and diagnosis from the full thickness skin graft group (FTSG-group) and the split thickness skin graft group (STSG-group).

a: scalp region, not otherwise specified

There was no difference between the two groups regarding distribution of the different dressing-materials. Data regarding healing and complications are shown in Table 3. In the FTSG-group*, *22 (78.6%) of the patients had a graft take of 90-100% at the date of unpacking of the dressing, 4 (14.3%) of 60-90%, 1 (3.6%) of 30-60% and 1 (3.6%) of <30%. Two patients (7.1%) had full necrosis of the skin graft with one of them undergoing a second skin grafting and the other one was left to secondary healing (Table 3). 

**Table 3:** Healing and complications

	**FTSG-group** **(n=28) (%)**	**STSG-group** **(n=78) (%)**	***p*** ** value**
Recipient site			
% of adherence			
90-100	22 (78.6)	64 (82.1)	0.75
60-90	4 (14.3)	6 (7.7)	
30-60	1 (3.6)	4 (5.1)	
<30	1 (3.6)	4 (5.1)	
Surgical complications			
Full loss of skin transplant	2 (7.1)	2 (2.6)	0.28
Retransplantation	1 (3.6)	1 (1.3)	0.46
Secondary healing	1 (3.6)	1 (1.3)	0.46
Reoperation (not full loss)	0 (0)	1 (1.3)	1.00
Infection treated with antibiotics	2 (7.1)	8 (10.3)	1.00
Hematoma	1 (3.6)	2 (2.6)	1.00
Seroma	0 (0)	5 (6.4)	0.32
Overall complication rate	5 (17.9)	18 (23.1)	0.79
Donor site			
Checked by a general practitioner or nurse health visitor	19 (67.9)	49 (62.8)	0.82
Surgical complications			
Necrosis	0 (0)	1 (1.3)	1.00
Wound rupture	1 (3.6)	0 (0)	1.00
Infection treated with antibiotics/flamazine/biatain Ag	1 (3.6)	4 (5.1)	1.00
Hematoma	0 (0)	1 (1.3)	1.00
			
Hospital stay (days, range)	0 (0-2)	0 (0-4)	0.10
Re-hospitalization (days, range)	0 (0-0)	0 (0-11)	0.55
Missing information		1	
Number of outpatient visits (no, range)	1 (1-14)	2 (1-14)	0.43
Missing information	1	2	
Time from operation until last outpatient visit, recipient site/”healing” (days, range)	7 (4-191)	10.5 (4-330)	0.97
Missing information	1	2	
Time from operation until last outpatient visit/”healing”, donor site (days, range)	6 (4-16)	14 (11-56)	**0.001**
Missing information	21	55	

Healing and complications for the full thickness skin graft group (FTSG-group) and the split thickness skin graft group (STSG-group) after scalp surgery

Two patients (7.1%) were treated with antibiotics due to postoperative infection. One patient developed a hematoma. In the STSG-group*,* 64 (82.1%) of the patients had a graft take of 90-100%, 6 (7.7%) of 60-90%, 4 (5.1%) of 30-60% and 4 (5.1%) of <30%. Two (2.6%) of the patients had a full necrosis of the skin graft with 1 (1.3%) of them undergoing a second skin grafting and 1 (1.3%) left to secondary healing. Eight (10.3%) of the patients were treated with antibiotics due to postoperative infection. 

Two (2.6%) patients developed a hematoma and 5 (6.4%) a seroma. There was no significant difference between the two groups regarding graft adherence and surgical complications. Overall complication rate was 5 (17.9%) in the FTSG-group and 18 (23.1%) in the STSG-group (*p=*0.79). At the donor site, one patient in the FTSG-group had wound dehiscence. One (3.6%) in the FTSG-group had infection of the donor site treated with antibiotics as opposed to 4 (5.1%) in the STSG-group*, *who were treated with Flamazine or Biatain Ag dressing. 

In the STSG-group*,* there was registered that one patient (1.3%) developed a hematoma and one had partial necrosis of the donor site. There was no statistically significant difference in donor site complications between the two groups. Duration of hospital stay had a median of 0 (0-2) days in the FTSG-group and 0 (0-4) in the STSG-group (*p*=0.10). Duration of re-hospitalization had a median of 0 (0-0) days in the FTSG-group and 0 (0-11) in the STSG-group (*p*=0.55). The number of outpatient visits was 1 (1-14) and 2 (1-14) in the FTSG-group and the STSG-group*, *respectively (*p*=0.43). Time from surgery until the last outpatient visit for wound care was 7 days (4-191) in the FTSG-group and 10.5 days (4-330) in the STSG-group (*p*=0.97).

## DISCUSSION

In the present study there were no significant statistical difference regarding preoperative data, adherence of the skin graft, complications or overall complication ratio. There was a significant difference concerning length and depth of the excision and also regarding the time from surgery until the last outpatient visit of the donor site, but not for the recipient site. We found a significant difference with the longest excision diameter in the split-thickness group and a larger percentage with deeper excision, to the pericranium, in the split-thickness skin graft group. 

This is most likely a matter of confounding by indication: larger size defects call for larger skin grafts and STSG would more often be chosen in such cases. Deeper defects can be at risk of more healing problems due to a less vascularized wound bed, and in such cases most surgeons would probably chose STSG over FTSG. The pericranium is though well vascularized, and no real contraindication for using a STSG.

The time from surgery until the last outpatient visit at the plastic surgery department could be seen as a proxy for the healing progress. We found no difference regarding the recipient site, but a significant difference regarding the donor site with a longer time for a split skin thickness graft. There were though missing data from over 50% of the patients in each group and most donor sites got controlled by the patients general practitioner. 

Wellington *et al.*^11^ found in their analysis of 31 patients with STSG and 16 with FTSG for coverage after radial forearm free flap harvest, no significant difference in time to healing at the donor site. Al Shlash *et al.*^12^ found in their study with 85 burn patients who received STSG (56 cases) and FTSG (29 cases) no significant advantage regarding graft failure, graft contraction, hyperpigmentation, altered sensation, infection rate and hospital stay.

In the process of deciding what graft to use potential complications, cosmetics of the donor and recipient site and patient satisfaction should all be considered, where the last part was not analyzed in this study. Hereafter, practical matters such as draping of the patient during surgery could also be determinant for the chosen graft and at last, with no difference in complications between the two groups, recommendations of which skin graft to use could also be based on the economical aspect; the costs of the surgery. 

Our main limitation is the retrospective nature of our study, leaving it vulnerable to confounding by indication, i.e. the surgeons could have been choosing STSG for complicated cases. If that was the case, STSG could be superior to FTSG. However, this cannot be determined from the present study. Furthermore, most patients were followed up at their General Practitioner concerning their donor site, from where we do not have data. However, we expect these cases to be rather uncomplicated, otherwise they should have been referred back to the department. 

Finally, due to the small number of patients in our case series, rare complications might not have been seen. Cosmetics and patient satisfaction could be analyzed retrospectively, although, given the limitations of a retrospective study, only a randomized controlled study will be able to examine if the two procedures are really equal. In conclusion we could not find any significant difference regarding graft take with the use of either FTSG or STSG in scalp defects, which is reassuring. 

## CONFLICT OF INTEREST

The authors declare no conflict of interest.
